# Aftereffects in face processing

**DOI:** 10.3389/fpsyg.2013.00854

**Published:** 2013-11-14

**Authors:** Peter J. Hills

**Affiliations:** Department of Psychology, Anglia Ruskin UniversityCambridge, UK

**Keywords:** face identity aftereffects, face distortion aftereffects, familiarity, adaptation, psychological, expression

The original aim of this special issue was to use aftereffects to highlight the different cognitive, perceptual, and neural representations of unfamiliar and familiar faces. Face aftereffects occur due to prolonged exposure to an adaptor face that causes a test face to take on the “opposite” characteristics (e.g., a normal face will appear compressed following adaptation to an expanded face, Webster and MacLin, 1999). The resulting papers went beyond this aim and have demonstrated the extensive potential for theoretical advancement that research on aftereffects can create.

Within the papers contained in this research topic is a highly informative review article. Strobach and Carbon (2013) have highlighted three dimensions that can be used as a framework to consider face aftereffects: adapting information (the type of adapting feature); temporal facets (including the duration that the aftereffect lasts); and transferability (across images and viewpoints). This framework necessarily implies the face distortion aftereffect (FDAE) and the face identity aftereffect (FIAE) are based same recalibration mechanism (Hills and Lewis, 2012). I will use this framework to guide this editorial.

By manipulating different facial features for adaptation, researchers have gone some way to understand the mechanismsof the adaptation process both specifically (in expressions) and in general. Dickinson and Badcock (2013) have shown that aftereffects in the perception of expressive faces (happy) is due to the angle of the mouth. Their novel conclusion is that aftereffects in expressions are due to the misperception of the orientation of the mouth due to the tilt aftereffect. This highlights the importance of ruling out lower-level explanations when considering face aftereffects, especially since aftereffects can occur at any level of the visual pathway (Thillman and Webster, 2012). More generally, Little et al. (2012) have shown that the FIAE is primarily based on face shape information rather than color information.

In one of the most inovative studies in face aftereffects, Vakli et al. (2012) have shown that the FDAE can be caused by gray stimuli with white dots in the triangular configuration of the internal facial features. This indicates that higher-level visual areas involved in the processing of facial configurations mediate the FDAE. Further evidence for the higher-level nature of facial aftereffects comes from evidence that shows there is residual sensitivity in the fusiform gyrus and the occipital face area in participants with acquired prosopagnosia (Fox et al., 2013). Furthermore, human bodies can adapt orientation-independent face representations (Kessler et al., 2013) further indicating the multi-modal nature of face aftereffects (see e.g., Hills et al., 2010).

In the current research topic, Carbon and Ditye (2012) were the only authors who explored the effects of temporal factors on the face aftereffects. They provided further evidence for the long-lasting effects of FIAEs in famous faces. These effects lasted 7 days and were observed even if the participant was tested in a different context to where they were adapted.

In terms of the transferability of the face aftereffects, Keefe et al. (2013) have shown that trustworthy aftereffects transfer across different face identities and to opposite gender faces. This result, coupled with data suggesting that there is some degree of selectivity of aftereffects (Juricevic and Webster, 2012; Rooney et al., 2012), indicates that there are likely to be many face prototypes: one for every trait that can be adapted to.

A series of studies in this research topic also explored the differences in aftereffects between faces of different levels of familiarity. Both Walton and Hills (2012) and Rooney et al. (2012) showed that aftereffects transferred across faces of different levels of facial familiarity. Specifically, aftereffects transferred from unfamiliar and famous faces to personally familiar faces, but not between famous and unfamiliar faces. This indicates that the representation of unfamiliar faces is distinct to famous faces, but both share some similiarities with the representation of personally familiar and self faces. Finally, aftereffects in famous faces transfer across viewpoint and photographic negation but not across orientation (Hills and Lewis, 2012; Vakli et al., 2012) indicating the representation of familiar faces is more robust than unfamiliar faces.

This research topic has also identified a number of practical advances in the study of face aftereffects. Little et al. (2012) have shown that these aftereffects are equivalent for laboratory based studies and studies conducted on the internet. These authors also noted that the aftereffects were stronger during the earlier trials during the post-adaptation test.

Several of the studies reported in this research topic show that the face aftereffect is in part carried by low-level mechanisms, in which aftereffects are twice as large when the adaptor and test image match than when the images do not match (Hills and Lewis, 2012; Juricevic and Webster, 2012). However, beyond this low-level effects, there are aftereffects in expressions, trustworthiness, identity, and distortion demonstrated in this research topic. The advancements made by the studies in the research topic have reiterated Frisby's (1979) comment that aftereffects are the psychophysists microelectrode.
